# Supplementary material for: Modelling Skylarks (Alauda arvensis) to Predict Impacts of Changes in Land Management and Policy: Development and Testing of an Agent-Based Model
Source: PLoS One. 2013 Jun 6;8(6):e65803. doi: 10.1371/journal.pone.0065803 (PMC3675089; doi:10.1371/journal.pone.0065803)
Supplement: Supporting Information S4 — The skylark ODdox as a zipped archive. (ZIP) [file pone.0065803.s004.zip › Skylark_ODdox/class_sk_territories-members.html]

ALMaSS Skylark ODdox: Member List


ALMaSS Skylark ODdox
 2.0


- Main Page
- Related Pages
- Classes
- Files

- Class List
- Class Index
- Class Hierarchy
- Class Members

SkTerritories Member List

This is the complete list of members for SkTerritories, including all inherited members.

|  |  |  |
| --- | --- | --- |
| ClaimGrid(int x, int y, int range) | SkTerritories |  |
| DumpMapGraphics(const char \*a\_filename, Landscape \*a\_map) | SkTerritories | private |
| EvaluateAllTerritories(void) | SkTerritories |  |
| EvaluateHabitatN(skTTerritory \*a\_terr) | SkTerritories |  |
| EvaluateHabitatSquare(int xmin, int xmax, int ymin, int ymax, int NoPolygons) | SkTerritories | private |
| FemaleOccupy(int ref, Skylark\_Female \*Female) | SkTerritories |  |
| GetTerritoriesByDistance(int nx, int ny, vector< Point > \*alist) | SkTerritories |  |
| Grid | SkTerritories | private |
| IsExtGridPositionValid(int &x, int &y, int range) | SkTerritories |  |
| IsGridPositionValid(int &x, int &y, int range) | SkTerritories |  |
| IsValid(int nx, int ny) | SkTerritories |  |
| m\_for\_iter\_x | SkTerritories | private |
| m\_for\_iter\_y | SkTerritories | private |
| m\_hash\_size | SkTerritories | private |
| m\_poly\_seen | SkTerritories | private |
| m\_poly\_size | SkTerritories | private |
| m\_qual\_cache | SkTerritories | private |
| m\_qual\_cache\_filled | SkTerritories | private |
| m\_qual\_grid | SkTerritories | private |
| m\_qual\_grid\_signal | SkTerritories | private |
| m\_sim\_h\_div\_10 | SkTerritories | private |
| m\_sim\_w\_div\_10 | SkTerritories | private |
| NoTerritories | SkTerritories | private |
| Occupy(int ref, Skylark\_Male \*Male) | SkTerritories |  |
| PolyHeightData | SkTerritories |  |
| PolyRefData | SkTerritories |  |
| PolySizeData | SkTerritories |  |
| PolyTypeData | SkTerritories |  |
| PreCachePoly(int a\_poly) | SkTerritories |  |
| PreEvaluateHabitat(int a\_x, int a\_y, int a\_range\_x, int a\_range\_y) | SkTerritories | inlineprivate |
| PreEvaluateHabitatStripX(int a\_x, int a\_y, int a\_range\_x) | SkTerritories | inlineprivate |
| PreEvaluateHabitatStripY(int a\_x, int a\_y, int a\_range\_x) | SkTerritories | inlineprivate |
| PreEvaluateQualGrid(SkQualGrid \*a\_grid, int a\_x, int a\_y, int a\_width, int a\_height) | SkTerritories | private |
| PreFillQualCache(void) | SkTerritories | private |
| PreFillQualGrid(void) | SkTerritories | private |
| PreFillTerrPolyLists(skTTerritory \*a\_terr) | SkTerritories | private |
| PreMakeForIterator(int a\_min\_incl, int a\_max\_excl, int \*a\_iter, int a\_norm\_max\_excl) | SkTerritories | private |
| PrePoly2Qual(int a\_poly) | SkTerritories | inline |
| PrePolyNQual(int a\_poly, int \*a\_good\_polys) | SkTerritories | inline |
| PreProcessLandscape2(Landscape \*L) | SkTerritories |  |
| RemoveFemale(int ref) | SkTerritories |  |
| RemoveMale(int ref) | SkTerritories |  |
| SimH | SkTerritories | private |
| SimW | SkTerritories | private |
| SkTerritories(Landscape \*L) | SkTerritories |  |
| Split(int ref) | SkTerritories |  |
| Supply\_F\_Owner(int ref) | SkTerritories |  |
| Supply\_Owner(int ref) | SkTerritories |  |
| Supply\_quality(int ref) | SkTerritories |  |
| Supply\_size(int ref) | SkTerritories |  |
| Supply\_terr(int ref) | SkTerritories |  |
| Supply\_x(int ref) | SkTerritories |  |
| Supply\_y(int ref) | SkTerritories |  |
| SupplyIsNestValid(int ref) | SkTerritories | inline |
| SupplyNestPosition(int ref) | SkTerritories | inline |
| SupplyNoFemaleOccupied() | SkTerritories |  |
| SupplyNoMaleOccupied() | SkTerritories |  |
| SupplyNoTerritories() | SkTerritories |  |
| Territories | SkTerritories | private |
| TheLandscape | SkTerritories |  |
| Tick(void) | SkTerritories | inline |
| UpdateQuality() | SkTerritories |  |
| ~SkTerritories() | SkTerritories |  |


- Generated on Thu Jan 10 2013 13:15:37 for ALMaSS Skylark ODdox by
   1.8.1.1
